# Tilted Orientation of Photochromic Dyes with Guest-Host Effect of Liquid Crystalline Polymer Matrix for Electrical UV Sensing

**DOI:** 10.3390/s16010038

**Published:** 2015-12-29

**Authors:** Amid Ranjkesh, Min-Kyu Park, Do Hyuk Park, Ji-Sub Park, Jun-Chan Choi, Sung-Hoon Kim, Hak-Rin Kim

**Affiliations:** 1 School of Electronics Engineering, Kyungpook National University, Daegu 702-701, Korea; amid@ee.knu.ac.kr (A.R.); mkpark@ee.knu.ac.kr (M.-K.P.); jspark19@ee.knu.ac.kr (J.-S.P.); jcchoi@ee.knu.ac.kr (J.-C.C.); 2 Department of Sensor and Display Engineering, Kyungpook National University, Daegu 702-701, Korea; parkdohyuk@gmail.com; 3 Department of Textile System Engineering, Kyungpook National University, Daegu 702-701, Korea; skokim@knu.ac.kr

**Keywords:** UV-sensing, spirooxazine (SO), photochromic dye, liquid crystal, metal-insulator-metal (MIM) structure, guest-host effect

## Abstract

We propose a highly oriented photochromic dye film for an ultraviolet (UV)-sensing layer, where spirooxazine (SO) derivatives are aligned with the liquid crystalline UV-curable reactive mesogens (RM) using a guest-host effect. For effective electrical UV sensing with a simple metal-insulator-metal structure, our results show that the UV-induced switchable dipole moment amount of the SO derivatives is high; however, their tilting orientation should be controlled. Compared to the dielectric layer with the nearly planar SO dye orientation, the photochromic dielectric layer with the moderately tilted dye orientation shows more than seven times higher the UV-induced capacitance variation.

## 1. Introduction

Several types of ultraviolet (UV) sensors have been proposed to measure doses of UV irradiation with greater efficiency and easier methods. In many cases, UV sensors have been developed based on the principles of photoconductive effects using several kinds of wide-band-gap inorganic materials, such as GaN, AlN, SnO_2_, and ZnO compounds [1,2,3,4,5]. Nanocrystalline diamond thin film [6] has been additionally investigated as an effective UV-sensing material [7]. For development into thin-film electronic devices, these inorganic materials require elaborate semiconductor manufacturing processes, which incur high processing costs. By using UV-sensitive organic materials, these issues can be overcome. Consequently, photochromic dyes have recently attracted considerable research interest [8,9,10,11,12]. The photochromic organic materials in a solution state can be easily made into a thin electro-optic film with a spin-coating or printing process. The optical transmittance variation or spectral change of the UV-irradiated photochromic film can be simply sensed, even with naked eyes [13]. Moreover, the UV dose can be quantitatively measured by employing a pair of probing optical sources and an optical detector. Nevertheless, this approach makes the optical system bulky [14]. Recently, electronic device applications using a photochromic film for UV sensors have being developed by utilizing photochromic molecular changes involving a large dipole moment variation or dielectric variation, which enables a quantitative measurement with a compact electronic device scheme [15,16,17].

Many types of photochromic dyes exist that can be used for UV detection [17,18,19,20]. Among organic photochromic compounds, spirooxazine (SO) dye is one of the most promising photochromic materials on account of its excellent light-fatigue resistance, photo-stability, large dipole moment change after UV irradiation, high sensitivity, and relatively fast response and recovery times [15,21,22]. The SO material is advantageous to UV-sensor applications with an electronic method [23,24,25] because its dipole moment can be remarkably changed from 1.62 D to 6.64 D after UV irradiation. In the case of spiropyran (SP) dye, as a comparison, the dipole moment variation after UV irradiation is minimal from 6.44 D to 13.9 D [16,17]. The photo-switchable dielectric properties of the SO dye can be used for UV sensing with electrical measurements by introducing the photochromic layer as a UV-sensitive insulating layer in thin-film-transistor (TFT) [26,27,28] or metal-insulator-metal (MIM) electronic structures [29,30,31,32].

However, in conventional electronic UV sensor approaches, the photochromic dyes have been used mixed with other dielectric polymer host materials just for improving the coating process [22,33]. In this case, the photochromic dye orientation within an insulating film is random, which means we cannot effectively utilize the UV-induced dipole change of the photochromic molecule. To improve the UV sensor based on photochromic organic molecules, we must control the molecular orientation of the dyes with consideration of their optical cross-sections for more effective UV absorption. We must additionally account for the molecular dipole direction along the electric field applied in the film for more effective electrical measurement.

In this work, we propose a method of developing a UV sensor that can quantitatively and effectively measure an incident UV intensity with an electrical method through the simple MIM structure. The MIM structure has a photochromic dielectric layer comprised by mixing the SO dyes into a UV-curable liquid crystalline reactive mesogen (RM). Conventionally, the RM molecules have been used for optical retardation films for liquid crystal (LC) displays to improve viewing angle properties [34,35] or patterned polarization-changing films for three-dimensional displays [36,37,38]. Here, by utilizing the guest-host effect in the mixed film, we demonstrate that the SO dyes as a guest material can be well-oriented by following the orientation of the liquid crystalline RM molecules used for the host material. After polymerization of the RM molecules with UV irradiation, the well-oriented guest-host film can be made into a physically stable solid dielectric film, which enables the capacitive measurement with the MIM structure to be applied.

The relationships between the effective tilting angles of the SO dye orientation determined by the RM layer and the UV sensing properties from the measured capacitance variation are optically and electrically investigated. To control the effective tilting angle, the polar anchoring property of the alignment layer is modified by introducing RM molecules into the conventional vertical alignment polyimide (PI) layer [39,40]. The UV-dependent capacitive variation of the MIM device can be improved by optimizing the tilting angle orientation of the photochromic SO molecules. Finally, we show that the polarization state of a UV incident can be detected by utilizing a polarization-dependent dipole change amount in our highly oriented photochromic film, which shows the possibility of novel sensing applications.

## 2. Operation Scheme and Experimental Procedure

### 2.1. Materials and Film Preparation

**Figure 1:**
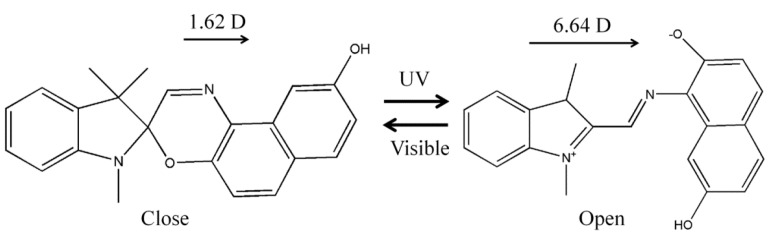
Photochromic molecular transformation of SO derivative dye and its dipole moment change under UV irradiation.

In our experiment, we employed the SO dye, which is a photochromic material with a large dipole moment change after UV irradiation, as mentioned above. As shown in Figure 1, the photo-cleavage of the C–O spiro bond occurs upon exposure to UV light. The subsequent molecular rotation around the C–C bond produces an open structure in the SO dye that is comprised of merocyanines (MCs) [41,42,43,44]. The MCs can thermally or photochemically return to the initial spiro form [23,42]. The MC has an extremely large dipole moment owing to its ionic form, while the SO dipole moment is relatively small. Therefore, the reversible photochromic molecular transformation of the SO derivative dye accompanied by a large dipole change enables quantitative UV detection with an electrical method [15,42].

In previous research, the photochromic dyes were used only when mixed with a polymer matrix to comprise a thin film as a gate insulator of TFTs or a dielectric layer of MIM devices. In our work, to obtain a highly oriented photochromic dye film, we used the guest-host method [23,43,44] by mixing the SO dyes (guest material) with the UV-curable liquid crystalline RM (host material). To dissolve the SO dyes into the RM, the RM solution (RMS03-013C, Merck, Darmstadt, Germany) was used. The mixing ratio of the SO dyes was 1 wt %. As a substrate, an ITO-coated glass substrate was used and an LC alignment PI layer was spin-coated on it. The alignment layer was unidirectionally rubbed with a rubbing machine to determine the azimuthal alignment direction of the RM host.

On the rubbed alignment layer, the solution mixed with the RM and photochromic SO dye was spin-coated at 4000 rpm for 30 s and dried for 1 min at 45 °C to remove the organic solvent within the mixture. After the drying process, the RM layer showed a nematic liquid crystalline phase with a highly orientational ordering. Depending on the polar and azimuthal anchoring conditions of the alignment layer, the orientation of the RM host was determined. The SO dye orientation likewise followed the same conditions as those of the guest molecule because the photochromic SO dye has a rod-like molecular shape, such as the RM shown in Figure 1. The initial rod-like molecular shape of the SO dye is one reason we selected it for use as a material from among the various photochromic dyes. Specifically, we aimed to utilize the effective molecular ordering of the dye doped in the RM host through the guest-host effect.

To obtain a solid film, the RM/dye mixture layer coated on the substrate was exposed by UV for 1 min at the intensity of 15 mW/cm^2^, where the polymerized RM film, in a SO-dye-mixed state, was produced via the photo-crosslinking process [45,46]. The thickness of the polymerized RM layer with the SO dyes was approximately 1.15 µm. The orientation of the dyes was well preserved after the polymerization of the host RM. To produce the photochromic change of the dyes aligned in the RM host film, a UV halogen lamp (*λ* = 365 nm) was used as an excitation optical source. For optical characterization of the photochromic change between coloration and discoloration depending on the orientation condition of the SO dyes, a He-Ne laser (*λ* = 633 nm) was used as a probe beam. The intensity of the He-Ne laser was reduced to 1 µW/cm^2^ to avoid the photochromic change induced by the probe beam.

### 2.2. Orientation Control with Alignment Layer

**Figure 2:**
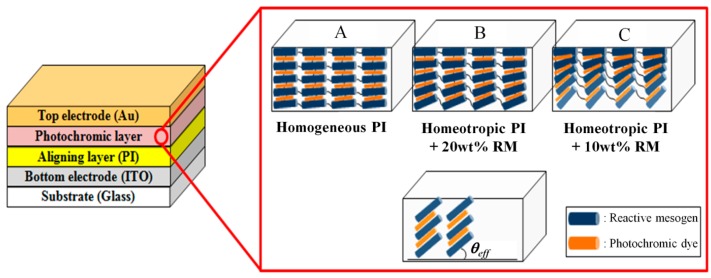
Schematics of the MIM structure for electrical measurement of UV intensity with the photochromic film. Tilting orientation angles of the photochromic dyes in the film are controlled by UV-cured liquid crystalline RM with the guest-host effect and polar RM anchoring conditions of the alignment layer.

Figure 2 shows our MIM device structure for electrical UV sensing utilizing a UV-sensitive photochromic dielectric layer. The azimuth orientation of the RM molecules and photochromic dyes follows the alignment direction of the PI layer, which is determined by the rubbing direction. In our system, the SO dyes are oriented by the host material (RM) via molecular interaction. The azimuth orientation angle for both molecules determined by the bottom PI surface is well preserved up to the top surface in our thin film. At the bottom surface, the polar orientation of the RM molecules and photochromic dyes is determined by the PI type, which can be planar or homeotropic to the surface, or even in an intermediate state, depending on the surface condition. However, before deposition of the top electrode, and during the UV irradiation process for the RM polymerization, the top surface condition of the RM layer is the air interface. At the ambient air interface, the polar orientation of the RM used in our experiment is planar.

Thus, when we use the planar LC anchoring PI as the bottom layer, the cross-linked RM network and the doped SO dyes show a homogeneously planar alignment without a tilting distribution along the depth, as shown in Case A of Figure 2. However, when the surface anchoring of the bottom PI layer is tilted, the polar orientation of the cross-linked RM and SO dyes linearly varies from the tilted angle at the bottom surface to the zero angle at the top surface on account of the elastic property of the LC, as shown in Figure 2 for Cases B and C. Even though the depth profile of the RM molecules and SO dyes continuously varies on the tilted anchoring surface, we simplify the structure and introduce the average effective tilting angle (*θ_eff_*), as shown in Figure 2, for characterizing the UV-sensitive optical and electrical properties of the dielectric layer.

In case A, the UV absorption efficiency the photochromic SO dyes and the rate of the photochromic molecular change will be most effectively compared to the other *θ_eff_* conditions with consideration of the molecular structure shown in Figure 1. However, the largest dipole moment change at the condition of *θ_eff_* = 0° cannot be effectively detected in the MIM structure shown in Figure 2 because the direction of the dipole moment and its change is perpendicular to the electrical field applied by two electrode plates. When increasing *θ_eff_*, the UV-induced dipole moment change of the SO dyes will be more effectively detected by the capacitance variation in the MIM structure. Instead, the effective optical cross-section of the dye molecules and UV absorption rate to the normally irradiated UV will be decreased. Therefore, to obtain an improved electrical UV-sensing property with the MIM structure, the optimum condition of *θ_eff_* should be determined in our experiment.

For the example of case A, a planar anchoring LC alignment PI (SE6514J, Nissan Chemical Industry, Tokyo, Japan) was used and spin-coated on the ITO glass for 30 s at 3000 rpm and baked for 30 min at 230 °C on a hot plate. For the samples with the tilted surface anchoring, mixtures of a homeotropic anchoring LC alignment PI (SE5661, Nissan Chemical Industry, Tokyo, Japan) and the RM solution (RMS03-013C, Merck, Darmstadt, Germany) were used. The PI/RM mixture solution was spin-coated on the ITO glass for 30 s at 3000 rpm. To polymerize the RM molecules doped in the homeotropic LC alignment PI, UV was radiated onto the spin-coated layer for 1 min at an intensity of 15 mW/cm^2^. Then, it was baked for 30 min at 220 °C on a hot plate for polymerization of the homeotropic LC alignment PI. To control *θ_eff_*, 10 wt% and 20 wt% of the RM doping density into the homeotropic LC alignment PI was used as shown in Figure 2 with Cases C and B, respectively.

Without doping the RM molecules, the tilting angle of the RM and SO dyes in the dielectric layer was 90° at the bottom homeotropic LC alignment PI layer. At this condition, the *θ_eff_* should have been the highest value considering the linearly varying depth profile of the polar angle [40,47,48]. However, on the homeotropic PI surface, the alignment property of the RM layer used as the dielectric layer was not good, even after the rubbing process, on account of the lower azimuth RM alignment property on the homeotropic PI [47,48,49,50,51]. By increasing the doping density of the RM molecules into the homeotropic PI, the tilting angle of the RM molecules and SO dyes in the dielectric layer decreased, along with the *θ_eff_* value, owing to the planar anchoring property of the polymerized RM molecules existing in the homeotropic PI layer. The surface tilting angle of the RM and SO molecules on the alignment layer was determined by anchoring the competition between the homeotropic anchoring PI and the planar-anchoring polymerized surface RM [51,52]. In addition, the alignment property of the RM molecules used in the dielectric layer could be improved by the well-aligned RM molecules within the PI layer by the rubbing process [40,48,49,50,51,52,53]. In our approach, the UV-cured surface RM molecules improved the alignment property of the bulk RM layer and controlled the *θ_eff_* property for efficient electrical UV sensing with the MIM structure.

### 2.3. MIM Structure for Electrical Measurement

As shown in Figure 2, to measure incident UV intensity with an electrical method, we prepared the MIM device by mixing the photochromic dyes into the RM dielectric layer. As the top electrode, 100 nm of the Au layer was thermally evaporated on the well-aligned RM/SO mixture dielectric layer at the slow deposition rate of 0.5 Å/s to avoid the Au penetration into the organic dielectric layer. An LCR meter (4284A; Agilent, Tokyo, Japan) was used to measure the electrical capacitance with a varying DC bias voltage in the range of −3 V~3 V at the fixed small-signal frequency condition of 1 MHz.

When we measured the UV-dependent capacitance variation with the MIM device with a homogeneously planar orientation of the SO dyes, such as in case A of Figure 2, the initial capacitance before the UV irradiation was approximately 16.9 nF/cm^2^. After exposing the UV light for 10 min, the measured capacitances for the different UV intensities of 1, 3, and 5 mW/cm^2^ were 17.1, 17.3, and 17.8 nF/cm^2^, respectively. There were no significant differences between the values of the capacitances, regardless of the radiated UV intensity. This finding shows that the planar orientation of the photochromic dyes is not appropriate for our electrical sensing scheme with the MIM structure, although the UV absorption efficiency would be the best at this condition.

## 3. Results and Discussion

### 3.1. Control of Photochromic Dye Orientation by Guest-Host Effect

**Figure 3:**
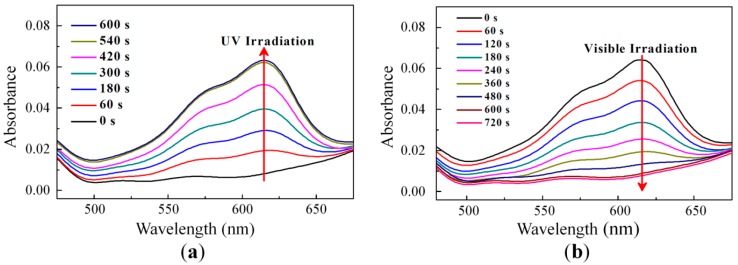
Absorption spectrum variation (**a**) under UV light irradiation; and (**b**) under visible light irradiation.

Figure 3 shows the absorption spectrum variation according to the UV light irradiation (*λ* = 365 nm with 5 mW/cm^2^) and the visible light irradiation measured with the homogeneously aligned RM/SO mixed film (such as Case A in Figure 2). As shown in Figure 3a, before the UV irradiation, the light transmittance of the film was high with a similar value over the whole visible regime. However, under the UV irradiation, the light absorption at approximately 633 nm continuously increased and was saturated. This result shows that the photochromic change of the film was due to the UV-induced molecular change, from the closed structure to the open structure, of the doped SO dyes. In addition, when we removed the UV irradiation onto the sample, the spectral light transmittance was continuously switched back to the initial one on account of the molecular change of the SO dyes to the ground state, as shown in Figure 3b. The UV-induced photochromic changing property of the dyes was highly dependent on the polarity of the adjacent polymer matrix condition [54]. Figure 3 shows that the UV-induced reversible photochromic switching behavior of the SO dyes doped into the liquid crystalline RM matrix was well preserved because the RM molecules were sufficiently cross-linked and changed into the chemically stable liquid crystalline polymer matrix by the UV irradiation processed during the photochromic dielectric layer preparation.

**Figure 4:**
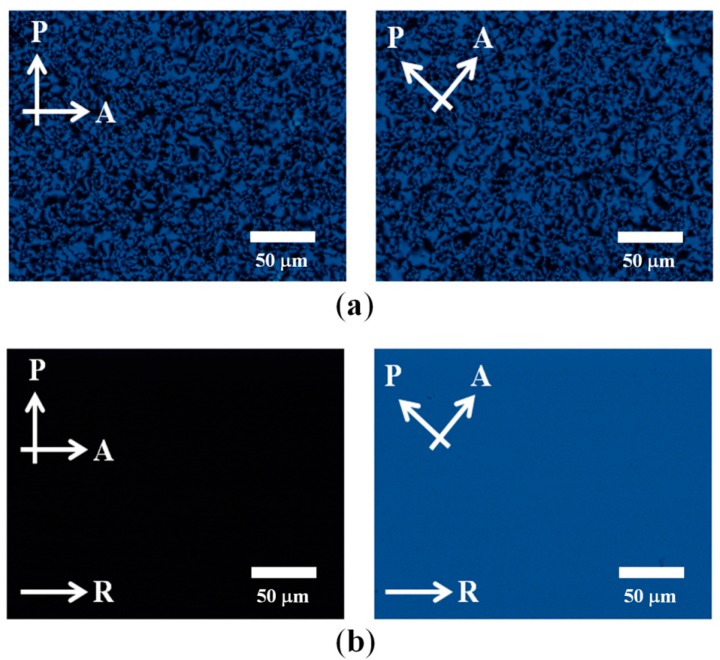
Polarizing optical microscopic images of guest-host films. (**a**) On a non-rubbed alignment layer; and (**b**) on a rubbed alignment layer. The arrows indicate the transmission axes of the polarizer (P) and analyzer (A), and the rubbing (R) direction of the alignment layer.

**Figure 5:**
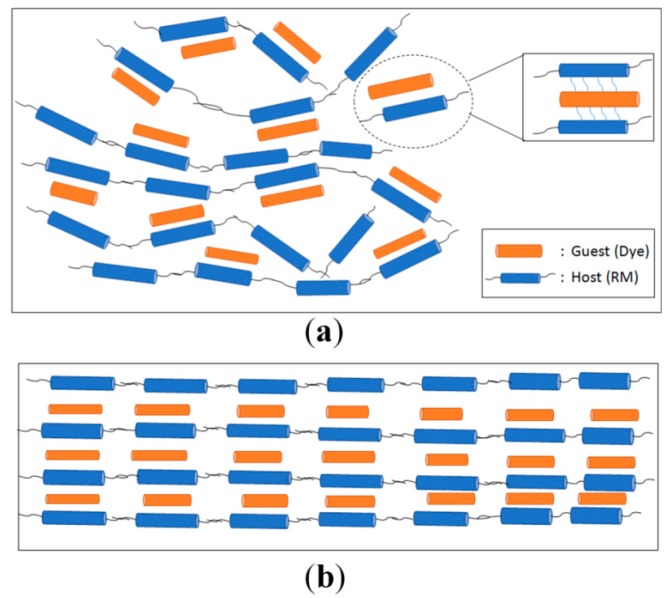
Schematic representation of the photochromic dye distribution determined by the UV-cured RM orientation with the guest-host effect. (**a**) Randomly distributed photochromic dyes on the non-rubbed alignment layer; and (**b**) uniformly distributed photochromic dyes on the rubbed alignment layer.

Figure 4 shows the polarizing optical microscope (POM) images of the photochromic guest-host film where the planar anchoring LC alignment PI was used for the RM alignment. In Figure 4a, the alignment layer is not rubbed; thus, the POM images show the schlieren textures [37] regardless of the sample rotation with respect to the crossed polarizers. This is because both the RM and SO dyes are not aligned by the surface condition, and the domains of the molecules are randomly distributed, as depicted in Figure 5a.

On the other hand, in the case of the POM images obtained with the sample prepared on the rubbed planar anchoring PI, completely dark and bright textures were obtained depending on the sample orientation with respect to the crossed polarizers, as shown in Figure 4b. Specifically, the dark texture was obtained when the rubbing direction of the PI was parallel to one of the crossed polarizers, indicating no deviation in average molecular orientation axis with respect to the rubbing direction. When the sample was rotated by 45° with respect to the polarizers, the uniformly bright texture was obtained. Both results indicate that the RM molecules were well aligned by the rubbed PI surface; moreover, the SO dyes were likewise well-aligned by the guest-host molecular ordering effect, as depicted in Figure 5b. To obtain the well-aligned photochromic dye properties, as shown in Figure 4b and Figure 5b, the initial rod-like molecular shape of the dye is important for utilizing the well-aligning effect of the rod-like RM molecule and its guest-host effect.

In the case of the sample prepared with the planar anchoring alignment PI, the photochromic molecular change should have been most efficient when considering the effective molecular cross-section area for the UV absorption. However, the dielectric change measured with the vertical field by the MIM structure was the smallest one because the direction of the dipole moment change was parallel to the molecular axis of the rod-like SO dye, as shown in Figure 1. It became orthogonal to the applied electric field direction.

### 3.2. UV Intensity Sensing by the Capacitance Variation Measurement

**Figure 6:**
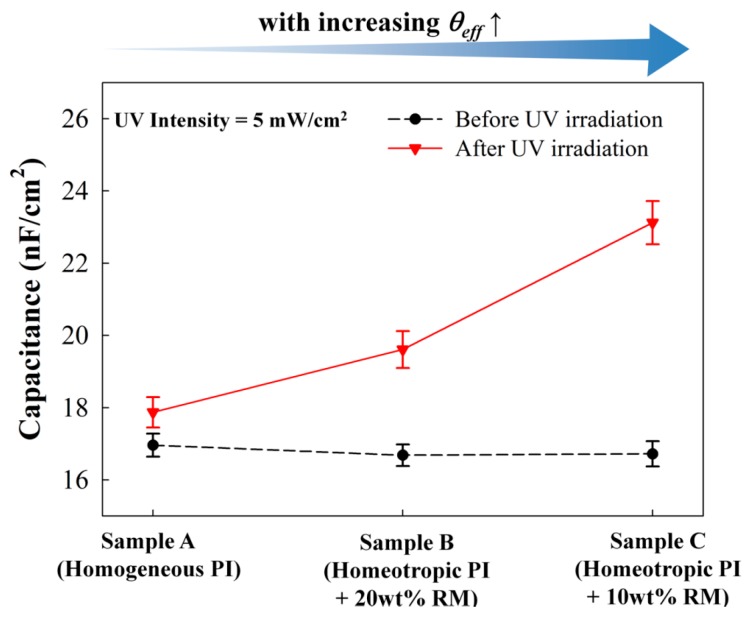
Capacitances of guest-host films with the MIM structure depending on the alignment layer conditions before and after UV irradiation.

To investigate the effect of the *θ_eff_* condition of the photochromic guest-host film on the electrical UV sensing, three types of the photochromic film with the MIM structure were prepared. For the reference condition, the MIM device with a photochromic guest-host dielectric layer aligned by the rubbed planar anchoring alignment PI was prepared, where the reference sample could be assumed as case A in Figure 2. For comparison, the photochromic guest-host dielectric layer with the highly tilted *θ_eff_* condition (<45°) were prepared with the RM-modified (10 wt% of the RM) homeotropic anchoring PI layer (Case C in Figure 2). As the intermediate *θ_eff_* condition (Case B in Figure 2), the MIM device with the photochromic guest-host dielectric layer aligned by the RM-modified (20 wt % of the RM) homeotropic anchoring PI layer was also prepared [39,40]. For each sample, the doping condition of the SO dyes into the RM host matrix was the same with 1 wt %. The thickness of the photochromic guest-host dielectric layer was carefully controlled to be a similar value of approximately 1.1 µm as shown in Figure 6 with the initial capacitance values (*C_o_*) before the UV irradiation.

Figure 6 shows the capacitances measured with each MIM device before and after UV irradiation. Hereafter, the UV irradiation condition for the photochromic change of the SO dyes was normal to the substrate. Before UV irradiation, the initial capacitances (*C_o_*) were approximately 16.9, 16.7, and 16.7 nF/cm^2^ for the samples of A, B, and C, respectively, which showed a minimal difference in the initial amount. For all samples, the UV irradiation conditions were 5 mW/cm^2^ for 10 min where the capacitance variations were almost saturated due to sufficient UV dose for photochromic molecular change irrespective the effective tilting condition of the SO dyes. After UV irradiation, the MIM capacitance of the sample A showed a slight change of Δ*C* = 0.9 nF/cm^2^. However, with an increase of the *θ_eff_* condition, the UV-induced capacitance variation significantly increased to Δ*C* = 2.9 and 6.4 nF/cm^2^ for the MIM samples of B and C, respectively. The UV-induced capacitance variation amount of the MIM device of the sample C with the highest *θ_eff_* condition was more than seven times higher than that prepared with the planar LC alignment layer (Sample A). This can be attributed to the fact that the direction of the UV-induced dipole moment change within the photochromic dielectric layer became more parallel to the electric field direction applied by the MIM structure. Moreover, the effective cross-section area of the SO dyes for the UV absorption was not notably decreased in our experimental RM-modified alignment conditions (*θ_eff_* < 45°). Note that the effective tilting angle of the SO dyes cannot exceed 45° in our tilted-planar RM geometry. However, the capacitance change to reach the saturated value was slower for the Sample C than that for the Sample A because of the reduced effective cross-section area of the highly tilted SO dyes.

**Figure 7:**
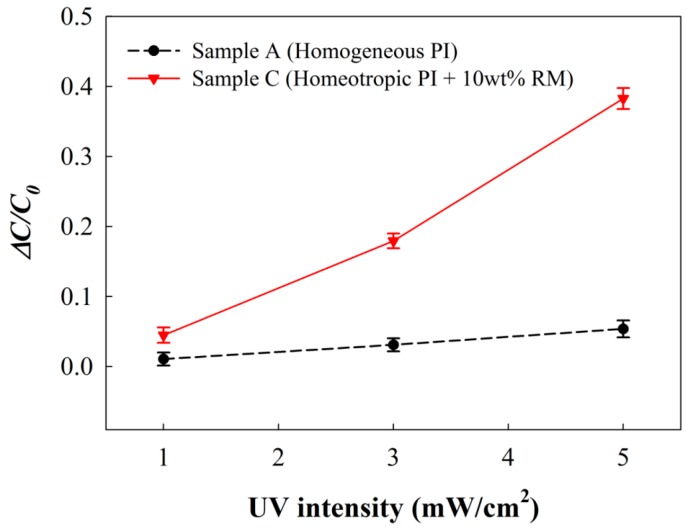
Relationship between UV sensitivity of the guest-host film in the electrical measurement and the alignment layer condition. Capacitance variation was measured with increasing UV irradiation intensities in the MIM samples of A and C.

When we evaluated the electrical UV intensity sensing property with the MIM structure dependency on the alignment layer condition, as shown in Figure 7, the amount of Δ*C/C_o_* in our MIM device was almost linearly increased with the increasing UV intensity for both samples of A and C. This result was due to the UV-induced increased molecular dipole moment of the SO dyes. However, the sensitivity was much higher in the MIM device of the sample C (with a higher *θ_eff_* condition) than the MIM device of the sample A. At the same UV irradiation condition of 5 mW/cm^2^ for 10 min, Δ*C/C_o_* for the sample C was approximately 0.38, whereas Δ*C/C_o_* for the sample A was only roughly 0.05.

In our work, for the electrical UV sensing with the photochromic dielectric layer, the MIM structure was used. However, the photochromic dielectric layer discussed here can also be applied to different types of electrical UV-sensing devices, such as organic and inorganic UV-sensing thin-film transistors (TFTs), as a UV-sensitive gate dielectric layer [16,55]. Compared to the MIM device presented here, the UV sensitivity can be significantly improved by adopting transistor structures. The UV-induced gate capacitance variation can result in a threshold-voltage-level shift, which could in turn result in the amplified-output-current-level variation [16,55,56]. However, in most cases of electrical sensing devices, the device structure is planar, such as the respective MIM and TFT structures; moreover, the electric field direction would be vertical. This means that our discussion on the UV-induced dielectric variation utilizing the photochromic effect of the organic dyes which is dependent on the tilting orientation and its control method is important for effectively producing the photochromic molecular change with UV absorption and effectively detecting the UV-induced molecular dipole change with an electrical method. The application of our approach to different types of electrical sensing devices is in progress.

### 3.3. Polarization-Dependent Capacitance Variation for UV Polarization Measurement

**Figure 8:**
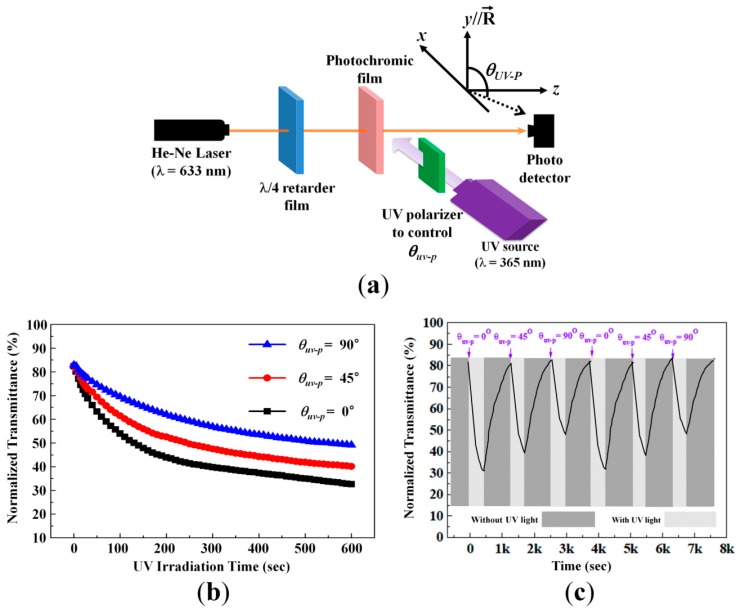
Photochromic properties of guest-host film depending on UV polarization. (**a**) Schematic diagram of the optical setup to measure the photochromic transmittance variation of the guest-host film in the visible range (probing beam: He-Ne laser) depending on the polarization condition (*θ_UV-P_*) of the UV irradiation; (**b**) Normalized transmittance of the photochromic guest-host film depending on *θ_UV-P_* of the UV irradiation; (**c**) Repetitive and reversible photochromic switching characteristic measured by changing *θ_UV-P_* of the UV irradiation.

In our photochromic dielectric layer, the RM molecules and SO dyes are azimuthally aligned along the rubbing direction of the alignment PI layer. This means that the UV-induced photochromic change is highly dependent on the polarization state of the UV irradiation. This effect has not been explored yet. First, to investigate the photochromic property that depends on the polarization state of the UV irradiation, we prepared an optical setup, as shown in Figure 8a.

For this experiment, we used the photochromic dielectric film with the sample C condition. All sample preparation conditions were the same as previously presented. The rubbing direction of the alignment layer was along the y-axis, as shown Figure 8a. The relative angle of the UV polarization with respect to the alignment direction of the photochromic film was denoted by *θ_UV-P_*. In the previous experiments, we used the non-polarized UV source for producing the photochromic change. However, for this experiment, the incident polarization was linearly polarized and its angle was changed by the UV polarizer. Because the UV source was non-polarized, the UV intensity irradiated onto the photochromic film was not changed at different *θ_UV-P_* conditions controlled by rotating the transmission axis of the UV polarizer. After the UV polarizer, the UV intensity was 5 mW/cm^2^ for this experiment. For the probe beam to check the photochromic change, the He-Ne laser was used after the intensity was sufficiently weakened. Before irradiating the probe beam, its polarization state was changed into circularly polarized light with the *λ*/4 retarder, as shown in Figure 8a. This was performed to avoid the polarization-dependent photochromic measurement for the probe beam.

Figure 8b shows the light transmittance of the probe beam under UV irradiation at different incident polarization conditions of *θ_UV-P_*. The light transmission change at the UV irradiation condition of *θ_UV-P_* = 0° is higher than that of *θ_UV-P_* = 90° because the UV absorption and resultant photochromic change are more efficient when the polarization direction of the UV, used as the molecular excitation beam, is parallel to the longitudinal direction of the SO dye, specifically when considering the molecular structure depicted in Figure 1. For the *θ_UV-P_* = 45° condition, the light transmittance levels by the photochromic change show almost average values of the two light transmittances obtained from the *θ_UV-P_* = 0° condition and *θ_UV-P_* = 90° condition over the whole UV irradiation time range. This is reasonable considering that the *θ_UV-P_* = 45° condition corresponds to the sum of half the UV intensity at the *θ_UV-P_* = 0° condition and the other half of the UV intensity at the *θ_UV-P_* = 90° condition. In other words, this additionally corresponds to the non-polarized UV irradiation condition. In our aligned film, the UV-induced photochromic property exhibits dichroic behavior.

Figure 8c shows the repetitive photochromic change of our guest-host film measured by changing the *θ_UV-P_* condition at the fixed UV intensity of 5 mW/cm^2^ after the UV polarizer. The duration times with and without the UV irradiation are 420 s and 930 s, respectively, and each of the times for the different *θ_UV-P_* conditions are the same. With UV irradiation, all results show that the photochromic change is initially fast and then becomes slightly slow. Without UV irradiation, the light transmittances are well recovered to the initial value under the repetitive test of the UV irradiation and relaxation without the UV irradiation. At the same UV irradiation time, the amount of the transmittance variation is the highest for the UV polarization condition of *θ_UV-P_* = 0°. In this repetition test, the amount of photochromic change obtained with the *θ_UV-P_* = 45° condition is the same as the average value of those obtained with the UV polarization conditions of *θ_UV-P_* = 0° and 90° at the same UV irradiation time.

**Figure 9:**
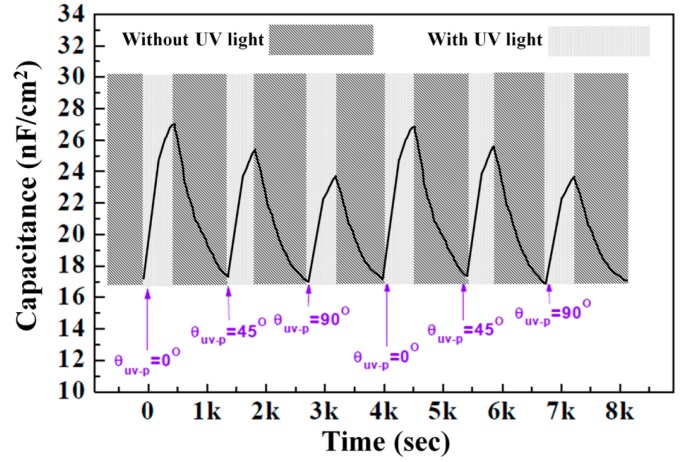
Electrical measurement of UV polarization with the guest-host film. Repetitive and reversible capacitance variation of photochromic guest-host films with the MIM structure measured by changing *θ_UV-P_* of the UV irradiation.

After preparing the MIM structure with the photochromic film used for Figure 8, we measured the repetitive capacitance variation by changing the *θ_UV-P_* condition of the UV irradiation. Figure 9 shows that the capacitance increases under the UV irradiation on account of the increased dipole moment of the SO dyes. Moreover, its variations are the highest and lowest when the UV polarization is respectively parallel and perpendicular to the molecular orientation of the photochromic dielectric layer. After removing UV irradiation, the capacitance well recovers to the initial value in our repetitive UV irradiation and relaxation test because the polymerized RM host matrix provides the chemically stable environmental condition for the reversible photochromic molecular change of the doped SO guest dyes. For the *θ_UV-P_* = 45° condition, the electrically measured amount of Δ*C* is the average value of those of *θ_UV-P_* = 0° and 90°, which is similar to the optical measurements of Figure 8. This can be attributed to the linear relationship between the UV intensity and capacitance variation in our MIM structure, as shown in Figure 7.

Utilizing this property of the capacitance variation that depends on the polarization state of the UV irradiation can enable new applications for the UV polarization measurement. In conventional approaches, to detect the polarization state of the UV irradiation, multiple sets of high-cost UV polarizers are used together with sets of UV detectors because the UV detector itself is polarization-insensitive. However, with our polarization-dependent photochromic film, the polarization state of an incident UV can be detected by preparing two MIM devices in a substrate, wherein the azimuthally molecular orientations of the two photochromic dielectric layers are aligned orthogonally to each other. Because the orientation of the SO dyes is determined by the RM orientation via the guest-host anisotropic molecular interaction, the conventional patterned PI alignment method can be easily used for this approach [45,46]. By using the linear capacitance variation property that depends on the incident UV intensity, along with the polarization-dependent capacitance variation, we can determine the UV-incident relative optical field components that are orthogonal to each other. This can be conducted after comparing the amounts of capacitance variations in the two MIM devices, which are prepared to enable the mutually orthogonal orientations of the photochromic dyes. For general applications that must detect the intensity level from the non-polarized UV, such as ambient UV, the polarization-sensitive photochromic property is not afflicted with issues, as shown in the results of Figure 6 and Figure 7.

## 4. Conclusions

In this work, we presented a UV-sensitive photochromic film in which rod-like SO dyes are well aligned by the liquid crystalline RM molecules via the molecular anisotropic interaction of the guest-host effect. By using the RM-modified alignment layer, the surface tilting condition of the RM molecules of the dielectric layer can be controlled, which results in the controlled bulk effective tilting angle of the SO dyes aligned by the bulk RM orientation. After optimizing the effective tilting angle of the photochromic layer, the UV intensity can be effectively measured with the electrical capacitance variation from the MIM device structure, wherein the photochromic layer is used as the UV-sensitive dielectric layer. Compared to the dielectric layer with the nearly planar SO dye orientation, the photochromic dielectric layer with the moderately tilted dye orientation prepared on the RM-modified anchoring surface shows more than seven times higher the capacitance variation at the same UV irradiation intensity condition. The capacitance variation is highly linear to the UV intensity before saturation. Within the UV-cured RM network, the reversible and repetitive photochromic molecular changes of the SO dyes that depend on the UV irradiation condition are quite stable.

Our approach of controlling the tilted orientation of the photochromic dyes with the guest-host effect can be widely applied to different types of electrical UV sensing devices on account of the enhanced measurable dipole moment change within the photochromic dielectric layer. In addition, we herein presented the possible application of the UV polarization measurement by utilizing the UV polarization-dependent photochromic property and its capacitance variation in our well-oriented photochromic film.
